# A heterogeneous response of liver and skeletal muscle fat to the combination of a Paleolithic diet and exercise in obese individuals with type 2 diabetes: a randomised controlled trial

**DOI:** 10.1007/s00125-018-4618-y

**Published:** 2018-04-26

**Authors:** Julia Otten, Andreas Stomby, Maria Waling, Andreas Isaksson, Ingegerd Söderström, Mats Ryberg, Michael Svensson, Jón Hauksson, Tommy Olsson

**Affiliations:** 1 Department of Public Health and Clinical Medicine, Division of Medicine, Umeå University, 90185 Umeå, Sweden; 2 Department of Food and Nutrition, Umeå University, Umeå, Sweden; 3 Department of Community Medicine and Rehabilitation, Sports Medicine Unit, Umeå University, Umeå, Sweden; 4 Department of Radiation Sciences, Radiation Physics and Biomedical Engineering, Umeå University, Umeå, Sweden

**Keywords:** Exercise, Hyperinsulinaemic–euglycaemic clamp, Insulin sensitivity, Intramyocellular fat, Liver fat, Nutrition, Obesity, Paleolithic diet, Proton magnetic resonance spectroscopy, Weight loss

## Abstract

**Aims/hypothesis:**

The aim of the study was to investigate ectopic fat deposition and insulin sensitivity, in a parallel single-blinded randomised controlled trial, comparing Paleolithic diet alone with the combination of Paleolithic diet and exercise in individuals with type 2 diabetes.

**Methods:**

Thirty-two individuals with type 2 diabetes with BMI 25–40 kg/m^2^ and 30–70 years of age followed a Paleolithic diet ad libitum for 12 weeks. In addition, study participants were randomised by computer program to either supervised combined exercise training (PD-EX group) or standard care exercise recommendations (PD group). Staff performing examinations and assessing outcomes were blinded to group assignment. Thirteen participants were analysed in each group: hepatic and peripheral insulin sensitivity were measured using the hyperinsulinaemic–euglycaemic clamp technique combined with [6,6-^2^H_2_]glucose infusion, and liver fat was assessed by proton magnetic resonance spectroscopy; both analyses were secondary endpoints. Intramyocellular lipid (IMCL) content was measured by magnetic resonance spectroscopy as a secondary analysis. All examinations were performed at Umeå University Hospital, Umeå, Sweden.

**Results:**

Both study groups showed a median body weight loss of 7 kg. Fat mass decreased by 5.7 kg in the PD group and by 6.5 kg in the PD-EX group. Maximum oxygen uptake increased in the PD-EX group only. Liver fat showed a consistent reduction (74% decrease) in the PD group, while the response in the PD-EX group was heterogeneous (*p* < 0.05 for the difference between groups). IMCL content of the soleus muscle decreased by 40% in the PD group and by 22% in the PD-EX group (*p* < 0.05 for the difference between groups). Both groups improved their peripheral and adipose tissue insulin sensitivity, but not their hepatic insulin sensitivity. Plasma fetuin-A decreased by 11% in the PD group (*p <* 0.05) and remained unchanged in the PD-EX group. Liver fat changes during the intervention were correlated with changes in fetuin-A (*r*_S_ = 0.63, *p <* 0.01). Participants did not report any important adverse events caused by the intervention.

**Conclusions/interpretation:**

A Paleolithic diet reduced liver fat and IMCL content, while there was a tissue-specific heterogeneous response to added exercise training.

**Trial registration:**

ClinicalTrials.gov NCT01513798

**Funding:**

Swedish Diabetes Research Foundation, County Council of Västerbotten, Swedish Heart and Lung Foundation, King Gustav V and Queen Victoria’s Foundation

**Electronic supplementary material:**

The online version of this article (10.1007/s00125-018-4618-y) contains peer-reviewed but unedited supplementary material, which is available to authorised users.

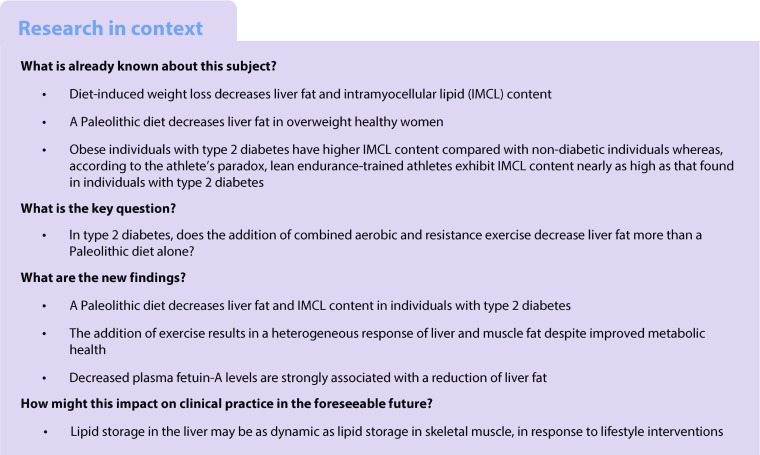



## Introduction

Fat accumulation outside adipose tissue, i.e. ectopic fat in liver and muscle, is linked to decreased insulin sensitivity and type 2 diabetes [1, 2]. However, intervention studies are needed to test a putative causal relationship between changes in ectopic lipid deposition and insulin sensitivity. Diet-induced weight loss in obese individuals is associated with reduction of fat in liver and skeletal muscle [3–5]. This has been linked to improved insulin sensitivity but has not been a universal finding. Notably, there are conflicting data regarding the effect of macronutrient composition on ectopic fat [3–7]. Two recent studies on obese postmenopausal women found that a Paleolithic diet consumed ad libitum with a moderately decreased carbohydrate intake and a high content of mono- and polyunsaturated fatty acids effectively reduced liver fat [8, 9]. Furthermore, a Paleolithic diet efficiently improved glucose tolerance in overweight individuals and in people with type 2 diabetes [10–12].

In contrast to the well-established relationship between diet-induced weight loss and decreased liver fat, it remains unclear whether exercise training decreases liver fat independently of weight reduction. An earlier intervention study in participants with type 2 diabetes reported that 4 months of either aerobic or resistance training was associated with a slight decrease in liver fat [13]. However, aerobic exercise combined with diet intervention does not appear to cause further liver fat reduction compared with diet intervention alone [14, 15]. To our knowledge, no prior study has investigated liver fat changes associated with diet in combination with both aerobic and resistance training. Interestingly, fetuin-A, a multifunctional protein secreted from both liver and adipose tissue, has been suggested as a putative link between insulin resistance and liver and adipose tissue function [16, 17].

Several studies show that individuals with obesity, insulin resistance and type 2 diabetes have higher intramyocellular lipid (IMCL) content compared with lean and healthy individuals [18, 19]. In obese individuals, weight reduction decreases IMCL content and simultaneously improves insulin sensitivity [20, 21]. However, lean endurance-trained athletes exhibit IMCL content measured at rest that is nearly as high as that of people with type 2 diabetes, but with concomitant normal insulin sensitivity, referred to as the athlete’s paradox [18]. Moreover, IMCL content is reduced immediately following an acute bout of aerobic exercise in young lean individuals [22]. This suggests that IMCL is an important intracellular source of energy during exercise in people with high insulin sensitivity. This dynamic response does not occur in obese individuals, who show unchanged IMCL content after 1 h of cycling [23]. Notably, a 12 week exercise intervention in individuals with type 2 diabetes found an increased IMCL content with concomitantly increased insulin sensitivity [24].

Owing to these inconclusive data, it is of major interest to study how a combination of diet intervention and aerobic and resistance training influences ectopic fat deposition and tissue-specific insulin sensitivity in individuals with type 2 diabetes. We therefore tested the hypothesis that overweight individuals with type 2 diabetes on a 12 week Paleolithic diet would exhibit a decrease in liver fat and IMCL content, associated with an improvement in hepatic and peripheral insulin sensitivity. Moreover, we hypothesised that combined aerobic and resistance exercise training would lead to a further improvement in liver fat and peripheral insulin sensitivity.

## Methods

### Study design

Overweight and obese individuals with type 2 diabetes consumed a Paleolithic diet for 12 weeks. In addition, the participants were randomised to receive either supervised exercise training for 3 h per week (PD-EX group) or standard care exercise recommendations (PD group). The reduction of fat mass was the primary endpoint of this study and has been published previously [25]. The outcome measurements of this article are secondary endpoints (liver fat and peripheral and hepatic insulin sensitivity) and secondary analyses (IMCL).

### Participants and randomisation

We used advertisements in local newspapers and posters at Umeå University Hospital, Umeå, Sweden, to recruit individuals with type 2 diabetes, age 30–70 years and BMI 25–40 kg/m^2^. For inclusion, participants were required to have an HbA_1c_ 48–95 mmol/mol (6.5–10.8%) and be treated with diet and/or metformin. Women had to be postmenopausal. Exclusion criteria were smoking, BP >160/100 mmHg, macroalbuminuria, cardiovascular disease, beta blocker use, severe illness and higher levels of training (e.g. moderate endurance training five times a week, resistance training every other week).

**Fig. 1:**
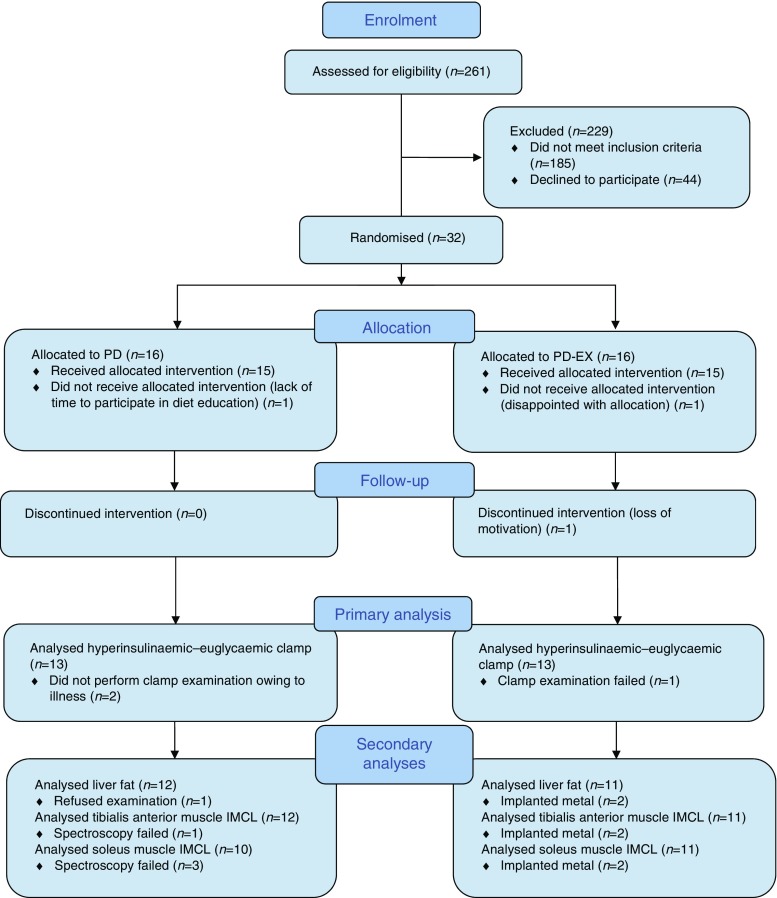
CONSORT flow diagram

A total of 261 individuals were assessed for eligibility, of whom 32 fulfilled the inclusion criteria and were randomised to the two groups (Fig. 1). Randomisation, using the computer program MinimPy version 0.3 [26], was performed by a statistician blinded to the study and not involved in data collection or analysis. Participants were assigned to either the PD group or the PD-EX group, using biased-coin minimisation with an allocation ratio of 1:1 [27]. The nurses and technicians who performed the examinations were blinded to group affiliation. All participants gave written informed consent. The study protocol was in accordance with the Helsinki Declaration and was approved by the Regional Ethical Review Board, Umeå, Sweden.

### Diet intervention

The Paleolithic diet included lean meat, eggs, fish, seafood, nuts, fruits and vegetables. Dairy products, cereals, legumes and added sugar and salt were excluded. Energy intake was ad libitum. Each study group attended five group sessions run by a trained dietitian, and participants could contact the dietitian by e-mail or phone between meetings. Dietary intake was assessed at baseline and at 12 weeks, using a 4 day self-reported weighed food record. A trained dietitian converted the food records into estimated energy and nutrient intakes using the nutritional calculation program Dietist XP 3.2 (Kost och Näringsdata, Bromma, Sweden).

### Exercise intervention

Prior to randomisation, all participants were advised to perform at least 30 min of moderate exercise daily in accordance with the current guidelines for people with type 2 diabetes. The PD-EX group additionally underwent a training protocol combining aerobic exercise and resistance training in 1 h sessions three times weekly at the Sports Medicine Unit at Umeå University, Umeå, Sweden. Low-intensity aerobic exercise was performed on a cross-trainer, and moderate- or high-intensity interval training was performed on a cycle ergometer. Resistance training included upper and lower body exercises involving multiple muscle groups. All exercise sessions were supervised by personal trainers with a Bachelor of Science degree in sports medicine.

### Body composition, liver fat, IMCL, $\overset{\cdot }{V}{\mathbf{O}}_{\mathbf{2max}}$**and energy expenditure**

Body composition analysis was performed using dual-energy x-ray absorptiometry (Lunar Prodigy X-ray Tube Housing Assembly, Brand BX-1L, Model 8743; GE Medical Systems, Madison, WI, USA) at the Clinical Research Centre at Umeå University Hospital. $\overset{\cdot }{V}{\mathrm{O}}_{2\max }$ was determined during a standard cardiopulmonary exercise test on a cycle ergometer at the Department of Clinical Physiology at Umeå University Hospital. Resting energy expenditure was measured by indirect calorimetry (Datex-Ohmeda Deltatrac II; Datex-Ohmeda, Madison, WI, USA) and adjusted by subtracting 5% during 8 h of sleep. Physical activity energy expenditure was estimated using a combined heart rate monitor and accelerometer for 7 consecutive days (Actiheart; CamNtech, Cambridge, UK) as previously described [28]. Total energy expenditure was calculated as the sum of resting energy expenditure and physical activity energy expenditure with added 10% for diet-induced thermogenesis.

Liver fat and tibialis anterior/soleus muscle fat were analysed by proton magnetic resonance spectroscopy as described in the electronic supplementary material (ESM) Methods.

### Insulin sensitivity and insulin clearance

Insulin sensitivity was assessed using the hyperinsulinaemic–euglycaemic clamp technique combined with [6,6-^2^H_2_]glucose infusion as previously described [24]. On the day of examination, participants came to the Clinical Research Centre at Umeå University Hospital in the fasted state having refrained from physical exercise for the prior 48 h. A catheter was placed in an antecubital vein for infusion. For blood sampling, a catheter was placed in the contralateral arm, retrograde into a superficial dorsal hand vein, with the hand placed in a heated box for arterialisation. Primed constant infusion of [6,6-^2^H_2_]glucose (APL, Stockholm, Sweden) at a rate of 0.22 μmol kg^−1^ min^−1^ was initiated at *t* = 0 min and continued until *t* = 360 min. At *t* = 180 min, we initiated primed constant infusion of short-acting insulin (Actrapid; Novo Nordisk, Bagsværd, Denmark) at a rate of 40 mU m^−2^ min^−1^, which continued until *t* = 360 min. Between *t* = 180 min and *t* = 360 min, blood was sampled every 5 min for immediate determination of plasma glucose concentration (HemoCue 201 RT; Radiometer Medical, Brønshøj, Denmark). Plasma glucose was clamped at 8 mmol/l by infusion of 20% glucose at a variable rate. This plasma glucose level was chosen based on previous hyperinsulinaemic–euglycaemic clamp studies [29, 30]. Arterialised blood was sampled for determination of plasma glucose and [6,6-^2^H_2_]glucose at *t* = 0, 150, 160, 170, 180, 330, 340, 350 and 360 min. We analysed plasma insulin at *t* = 0, 180, 240, 300 and 360 min, and NEFA at *t* = 0, 240, 270, 300, 330 and 360 min.

### Gene expression

Real-time quantitative PCR was used to determine relative gene expression of *TNFα* and *IL6* in subcutaneous adipose tissue of the abdomen (see ESM Methods for further details).

### Blood sample analysis

Blood samples were taken in the fasting state from a peripheral vein, followed by immediate analysis of plasma insulin, HbA_1c_, serum triacylglycerols, plasma aspartate aminotransferase (AST), plasma alanine aminotransferase (ALT) and plasma C-reactive protein (CRP) in the Clinical Chemistry unit at Umeå University Hospital. For NEFA analysis, plasma was stored at −80°C and later analysed using the NEFA-HR_2_ kit (Wako Chemicals, Neuss, Germany). Serum fetuin-A concentrations were determined using a human fetuin-A ELISA kit (BioVendor, Brno, Czech Republic).

The arterialised venous samples of the hyperinsulinaemic–euglycaemic clamp were analysed for [6,6-^2^H_2_]glucose and unlabelled glucose using GC-MS at the Swedish Metabolomics Centre, Umeå, Sweden. The samples (100 μl) were extracted with 90% methanol (900 μl), including ^13^C_6_-d-glucose as internal standard, then derivatised by addition of freshly prepared acetic anhydride/pyridine (1/1 vol./vol.). Next, the solvent was removed by a stream of N_2_, and thereafter the samples were dissolved in ethyl acetate. These samples were injected by an Agilent 7693 autosampler (Agilent Technologies, Atlanta, GA, USA) into an Agilent 7890A gas chromatograph, and analysed in an Agilent 7010C QQQ mass spectrometer operating in selected ion monitoring mode. The fragment ion m/z 244 was used to detect [6,6-^2^H_2_]glucose; m/z 242 was used for unlabelled glucose and m/z 247 for ^13^C_6_-d-glucose. To determine levels of [6,6-^2^H_2_]glucose and unlabelled glucose, calibration curves were set up between calibrants and internal standard.

### Calculations

Endogenous glucose production (EGP) was determined using Steele’s single-pool non-steady-state equation [31]. The glucose distribution volume was estimated as 0.1625 l/kg body weight. The suppression of EGP (%) was calculated as [EGP basal (*t* = 150, 160, 170, 180 min) − EGP clamp (*t* = 330, 340, 350, 360 min)] × 100 / EGP basal. To determine the rate of disappearance, the glucose infusion rate during the last 30 min of clamping was added to the EGP clamp and corrected for the non-steady-state condition using Steele’s equation. One participant was excluded from calculations of EGP and rate of disappearance because the [6,6-^2^H_2_] glucose infusion pump did not work properly during the third hour of the examination. Suppression of NEFA (%) was calculated as [(NEFA at *t* = 0 min) − (NEFA at *t* = 240, 270, 300, 330, 360 min)] − 100 / (NEFA at *t* = 0 min) [32]. Insulin clearance during insulin infusion was calculated by dividing the insulin infusion rate [mU (kg FFM)^−1^ min^−1^], where FFM is fat-free mass, by the mean plasma insulin concentration during insulin infusion [32].

### Statistical analysis

Several variables showed a skewed distribution; thus, we used the Wilcoxon rank-sum test to compare the treatment effect (change from baseline to 12 weeks) between the PD and PD-EX groups. The change over time within each intervention group was determined using the Wilcoxon signed-rank test. Correlation analyses were performed using Spearman’s rho (*r*_S_). A two-sided *p* value of <0.05 was considered statistically significant. All statistical analyses were performed using R, version 3.2.2, a language and environment for statistical computing (R Foundation for Statistical Computing, Vienna, Austria). Data are presented as median (interquartile range).

## Results

### Participant characteristics and intervention validation

The main results of the intervention have been previously published [25]. In the present substudy, we found no between-group differences in baseline characteristics except a higher fasting glucose in the PD-EX group (Table 1). Median weight loss was 7 kg in both groups (Table 1). Fat mass decreased by 5.7 kg in the PD group and by 6.5 kg in the PD-EX group (Table 1). Food records showed that both study groups similarly reduced total energy intake during the ad libitum diet intervention, mostly by decreasing intake of carbohydrates and saturated fatty acids (Table 2). $\overset{\cdot }{V}{\mathrm{O}}_{2\max }$ increased by 10% in the PD-EX group and decreased by 3% in the PD group (*p <* 0.01 for between-group difference). Total physical activity energy expenditure did not change in either group (Table 2).

**Table 1:** Baseline characteristics, body weight, and fasting blood samples

Variable	PD group	PD-EX group
*n* (male/female)	13 (9/4)	13 (8/5)
Age, years	60 (54, 64)	61 (58, 67)
Diabetes duration, years	3 (2, 6)	5 (1, 8)
Body weight, kg		
Baseline	90.0 (83.3, 103.2)	97.2 (82.9, 107.4)
Change 0–12 weeks	−7.1 (−9.8, −5.6)***	−7.0 (−9.7, −5.6)***
BMI, kg/m^2^		
Baseline	31.4 (29.4, 33.7)	31.4 (29.0, 34.6)
Change 0–12 weeks	−2.4 (−3.1, −1.8)***	−2.3 (−3.4, −2.2)***
Fat mass, kg		
Baseline	34.4 (30.1, 37.9)	33.6 (29.2, 39.2)
Change 0–12 weeks	−5.7 (−8.2, −4.0)***	−6.5 (−8.9, −5.1)***
HbA_1c_, mmol/mol		
Baseline	55 (48, 58)	56 (50, 59)
Change 0–12 weeks	−11 (−15, −5)**	−11 (−18, −7)**
HbA_1c_,%		
Baseline	7.2 (6.5, 7.5)	7.3 (6.7, 7.5)
Change 0–12 weeks	−1.0 (−1.4, −0.5)**	−1.0 (−1.7, −0.6)**
Fasting plasma glucose, mmol/l		
Baseline	8.0 (6.9, 8.5)	8.6 (7.7, 10.5)^†^
Change 0–12 weeks	−0.9 (−1.8, −0.1)*	−2.0 (−3.0, −1.0)**
Serum triacylglycerols, mmol/l		
Baseline	2.4 (1.4, 3.1)	1.7 (1.0, 2.3)
Change 0–12 weeks	−0.6 (−1.8, −0.2)*	−0.4 (−1.0, −0.1)**
Plasma NEFA, mmol/l		
Baseline	0.60 (0.53, 0.78)	0.80 (0.65, 0.90)
Change 0–12 weeks	0.03 (−0.02, 0.20)	−0.04 (−0.12, 0.17)
Plasma AST, μkat/l		
Baseline	0.59 (0.53, 0.71)	0.55 (0.51, 0.66)
Change 0–12 weeks	−0.05 (−0.14, 0.14)	0.02 (−0.20, 0.22)
Plasma ALT, μkat/l		
Baseline	0.67 (0.53, 0.91)	0.54 (0.48, 0.78)
Change 0–12 weeks	−0.12 (−0.34, −0.08)**	−0.07 (−0.16, 0.07)

Data are reported as median (interquartile range)

*p < 0.05, **p < 0.01, ***p < 0.001 for the within-group change over time from baseline to 12 weeks

^†^*p <* 0.05 between the PD and PD-EX groups

**Table 2:** Energy balance and dietary intake

Variable	PD group (*n* = 12)	PD-EX group (*n* = 12)
Energy intake, kJ/day		
Baseline	8330 (6204, 10,778)	6673 (5569, 9443)
Change 0–12 weeks	−1377 (−3284, −1025)**	−2155 (−3330, −649)**
Total energy expenditure, kJ/day		
Baseline	12,619 (11,129, 13,933)	12,485 (9196, 16,778)
Change 0–12 weeks	−954 (−1485, −285)*	−1305 (−2414, 1201)
Resting energy expenditure, kJ/day		
Baseline	6791 (6184, 7243)	7268 (5565, 7958)
Change 0–12 weeks	−510 (−774, −188)**	−381 (−715, −92)**
Physical activity energy expenditure, kJ/day		
Baseline	4276 (3615, 5519)	4201 (3008, 7201)
Change 0–12 weeks	−117 (−870, 372)	−88 (−1686, 1640)
Protein, g/day		
Baseline	80 (69, 95)	77 (63, 106)
Change 0–12 weeks	5 (−17, 23)	1 (−16, 14)
Protein, g kg^−1^ day^−1^		
Baseline	0.85 (0.66, 1.14)	0.78 (0.73, 1.02)
Change 0–12 weeks	0.10 (−0.09, 0.35)	0.06 (−0.12, 0.25)
Carbohydrate, g/day		
Baseline	204 (148, 280)	169 (152, 197)
Change 0–12 weeks	−89 (−122, −49)**	−92 (−117, −67)**
Total fat, g/day		
Baseline	84 (58, 115)	64 (46, 98)
Change 0–12 weeks	−12 (−38, 8)	−10 (−33, 24)
Saturated fatty acids, g/day		
Baseline	31 (21, 48)	25 (19, 35)
Change 0–12 weeks	−14 (−33, −5)**	−12 (−23, −6)**
Monounsaturated fatty acids, g/day		
Baseline	32 (25, 41)	26 (16, 37)
Change 0–12 weeks	4 (−16, 14)	5 (−6, 18)
Polyunsaturated fatty acids, g/day		
Baseline	11 (9, 14)	9 (7, 16)
Change 0–12 weeks	1 (−5, 5)	1 (−3, 7)
Protein, E%		
Baseline	17 (14, 19)	18 (17, 20)
Change 0–12 weeks	7 (4, 11)**	6 (3, 12)**
Carbohydrate, E%		
Baseline	41 (38, 46)	45 (32, 49)
Change 0–12 weeks	−10 (−18, −3)*	−14 (−21, −7)**
Total fat, E%		
Baseline	40 (36, 41)	32 (31, 44)
Change 0–12 weeks	6 (−6, 11)	9 (2, 14)*
Saturated fatty acids, E%		
Baseline	15 (13, 18)	13 (12, 17)
Change 0–12 weeks	−5 (−8, −3)**	−4 (−7, −2)**
Monounsaturated fatty acids, E%		
Baseline	16 (14, 17)	12 (10, 17)
Change 0–12 weeks	5 (−3, 11)	10 (5, 12)**
Polyunsaturated fatty acids, E%		
Baseline	5.0 (4.7, 6.4)	5.6 (3.8, 6.4)
Change 0–12 weeks	2.1 (−0.5, 3.6)*	3.2 (0.9, 3.7)**

Data are reported as median (interquartile range)

*p < 0.05, **p < 0.01 for the within-group change over time from baseline to 12 weeks

E%, energy per cent

### Insulin sensitivity and insulin clearance

**Fig. 2:**
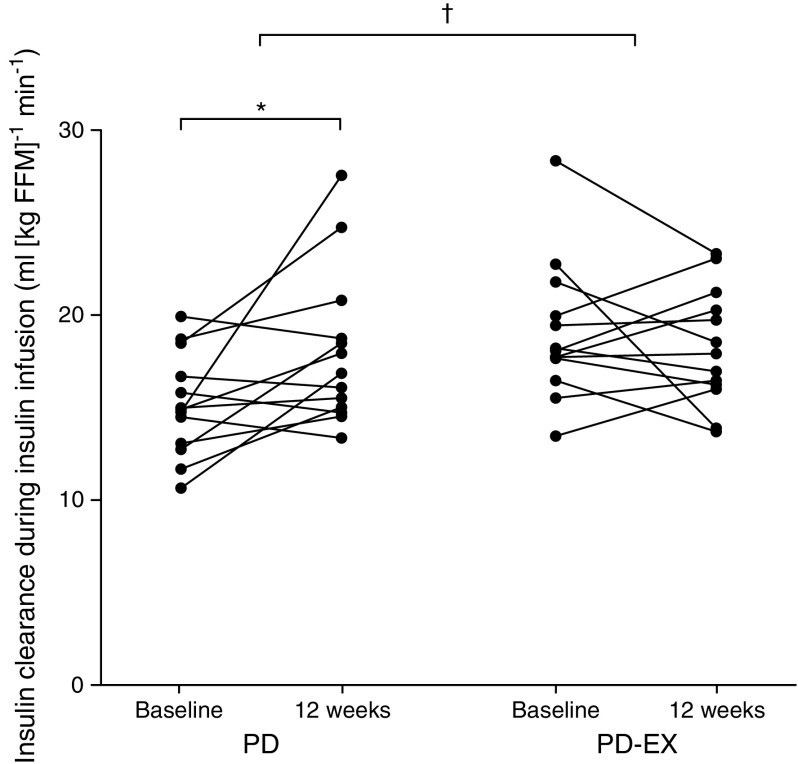
Insulin clearance during 12 weeks of intervention in the PD and PD-EX groups. *p < 0.05 for the within-group change over time from baseline to 12 weeks. ^†^*p <* 0.05 for the intervention effect between the PD group and the PD-EX group

Peripheral insulin sensitivity, measured as the rate of disappearance during the hyperinsulinaemic–euglycaemic clamp, increased by 53% in the PD group (*p <* 0.05) and by 42% in the PD-EX group (*p <* 0.01) (Table 3). Hepatic insulin sensitivity, measured as suppression of EGP, remained essentially unchanged during the intervention (Table 3). Both study groups showed increased NEFA suppression during the hyperinsulinaemic–euglycaemic clamp. Adipose tissue insulin sensitivity, calculated as NEFA suppression during the whole clamp examination, increased by 3.4% in both intervention groups (*p <* 0.05 in the PD group, *p <* 0.01 in the PD-EX group) (Table 3). When the insulin sensitivity measures were normalised for plasma insulin during the clamp, the PD group showed more pronounced improvement compared with the PD-EX group (Table 3). This was due to increased insulin clearance in the PD group (*p <* 0.05 for the between-group difference; Fig. 2). The intervention groups did not significantly differ in any measure of insulin sensitivity.

**Table 3:** Insulin sensitivity

Insulin sensitivity	PD group	PP-EX group
Peripheral insulin sensitivity		
Rate of disappearance, mg kg^−1^ min^−1^		
Baseline	3.79 (2.95, 4.23)	3.87 (3.02, 5.26)
Change 0–12 weeks	2.05 (0.32, 3.59)*	1.15 (0.67, 2.66)**
Rate of disappearance/insulin, μg kg^−1^ min^−1^ per mU/l		
Baseline	34.2 (28.6, 49.2)	46.2 (35.0, 74.5)
Change 0–12 weeks	28.9 (11.8, 61.5)**	14.6 (5.0, 30.3)**
Hepatic insulin sensitivity		
EGP, mg kg^−1^ min^−1^		
Baseline	1.81 (1.56, 1.99)	1.78 (1.51, 2.49)
Change 0–12 weeks	0.04 (−0.06, 0.55)	0.11 (−0.18, 0.60)
Suppression of EGP, %		
Baseline	96 (83, 128)	114 (85, 121)
Change 0–12 weeks	13 (−10, 44)	11 (−33, 35)
Suppression of EGP/insulin, % per mU/l		
Baseline	0.98 (0.77, 1.36)	1.51 (0.97, 1.74)
Change 0–12 weeks	0.22 (−0.03, 0.91)*	0.06 (−0.45, 0.55)
Adipose tissue insulin sensitivity		
Suppression of NEFA, %		
Baseline	88 (80, 93)	89 (85, 93)
Change 0–12 weeks	3.4 (1.3, 5.7)*	3.4 (0.2, 7.8)**
Suppression of NEFA/insulin, % per mU/l^1^		
Baseline	0.83 (0.74, 1.08)	1.12 (0.95, 1.27)^†^
Change 0–12 weeks	0.14 (0.03, 0.31)**	0.11 (−0.06, 0.20)

Data are reported as median (interquartile range)

*p < 0.05, **p < 0.01 for the within-group change over time from baseline to 12 weeks

^†^*p <* 0.05 between the PD and PD-EX groups

### Fat in soleus and tibialis anterior muscle

**Fig. 3:**
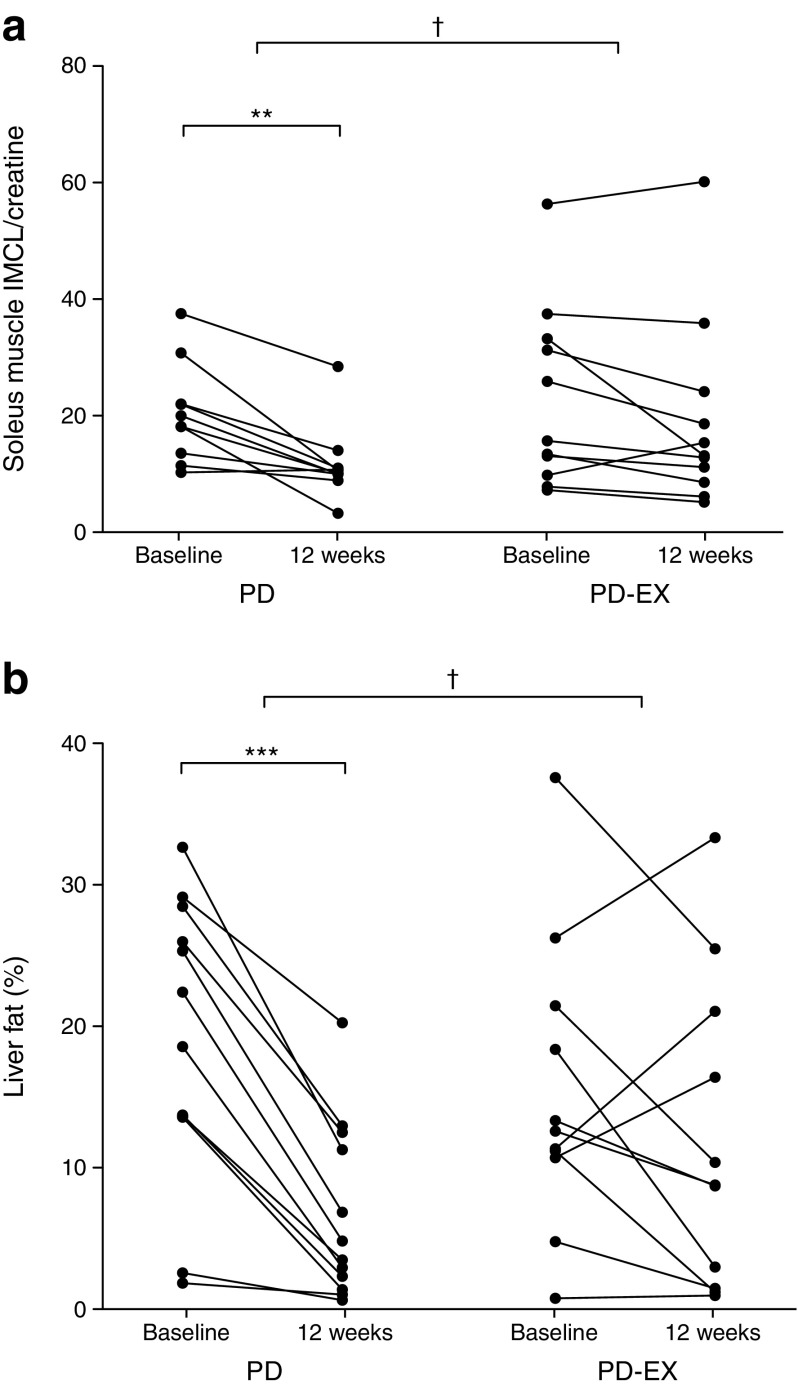
IMCL content of the soleus muscle (**a**) and liver fat (**b**) during 12 weeks of intervention in the PD and PD-EX groups. IMCL is normalised to the creatine concentration of the muscle. **p < 0.01, ***p < 0.001 for the within-group change over time from baseline to 12 weeks. ^†^*p <* 0.05 for the intervention effect between the PD group and the PD-EX group

IMCL content of the soleus muscle decreased by 40% in the PD group, but showed no significant change in the PD-EX group (Fig. 3a). Tibialis anterior IMCL content did not change significantly in either intervention group (data not shown). Tibialis anterior IMCL content at baseline was associated with peripheral insulin sensitivity (*r*_S_ = −0.45, *p <* 0.05) and fasting plasma NEFA (*r*_S_ = 0.59, *p <* 0.05). Changes in tibialis and soleus muscle IMCL content during the intervention were not correlated with changes in peripheral insulin sensitivity.

### Liver fat

All participants in the PD group decreased their liver fat, while the intervention response was more heterogeneous in the PD-EX group (Fig. 3b). Median hepatic lipid reduction was 74% for the PD group and 32% for the PD-EX group (*p <* 0.05 for the difference between groups).

Liver fat increased substantially in three individuals in the PD-EX group. After exclusion of these outliers, the median decrease in liver fat in the PD-EX group was 43% (*p <* 0.05), with no significant difference between the intervention groups (*p* = 0.08). Excluding the three outliers did not alter the results of the other statistical analyses (ESM Table 1). The three individuals whose liver fat increased during the intervention had a greater decrease in soleus muscle IMCL content compared with the other participants in the PD-EX group (ESM Table 1). $\overset{\cdot }{V}{\mathrm{O}}_{2\max }$, plasma triacylglycerols, plasma AST, plasma ALT and plasma CRP were improved or unchanged in these three individuals (ESM Table 1).

Liver fat was not associated with hepatic insulin sensitivity at baseline. The decrease in liver fat during the intervention was correlated with an improvement in hepatic insulin sensitivity in the PD group (*r*_S_ = −0.62, *p <* 0.05) but not in the PD-EX group. Liver fat was associated with adipose tissue insulin sensitivity (NEFA suppression during the clamp) at baseline (*r*_S_ = −0.58, *p <* 0.01).

### Plasma fetuin-A

**Fig. 4:**
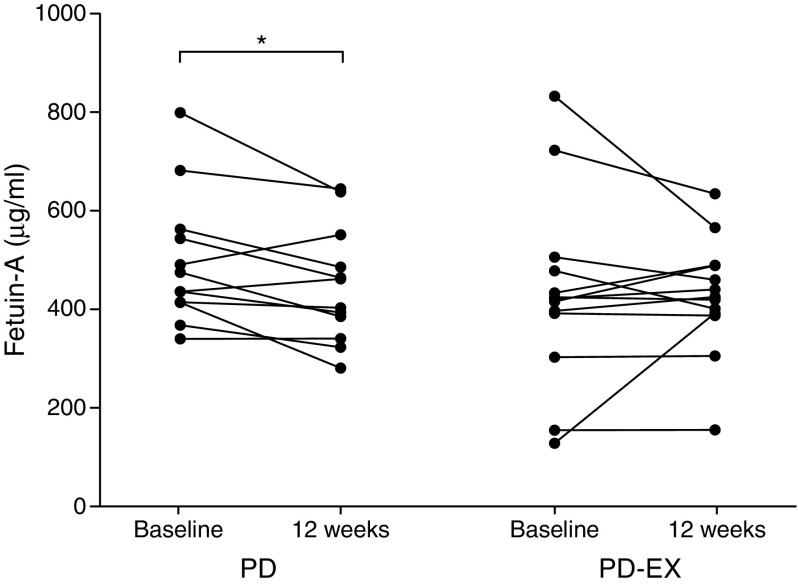
Plasma fetuin-A levels during 12 weeks of intervention in the PD and PD-EX groups. *p < 0.05 for the within-group change over time from baseline to 12 weeks

**Fig. 5:**
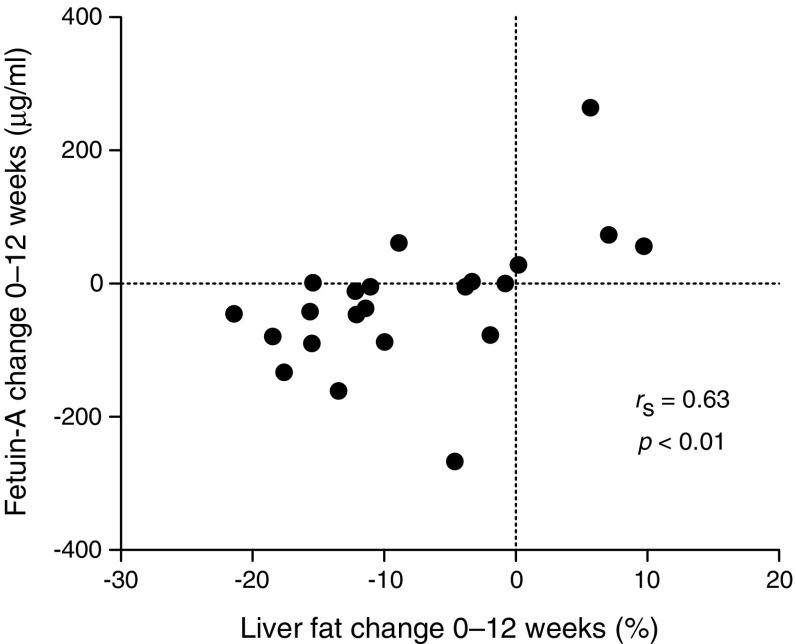
Association between change in liver fat and change in plasma fetuin-A during the intervention

Plasma fetuin-A decreased by 11% in the PD group (*p <* 0.05) and remained unchanged in the PD-EX group (Fig. 4). Liver fat changes during the intervention were strongly correlated with changes in fetuin-A (*r*_S_ = 0.63, *p <* 0.01; Fig. 5). Changes in adipose tissue insulin sensitivity (suppression of NEFA/insulin) were also associated with changes in fetuin-A (*r*_S_ = 0.51, *p <* 0.01). By contrast, we found no association between changes in fetuin-A levels and changes in hepatic or peripheral sensitivity. The three participants in the PD-EX group who showed increased liver fat also showed increased plasma fetuin-A levels (Fig. 5).

### Inflammation in plasma and adipose tissue

Plasma CRP decreased in the PD-EX group (*p <* 0.05; Table 4). In adipose tissue, *IL6* and *TNFα* gene expression did not change significantly during the intervention. During the intervention, the change in *TNFα* gene expression in subcutaneous adipose tissue was associated with the change in fasting plasma NEFA (*r*_S_ = 0.45, *p <* 0.05).

**Table 4:** Inflammatory markers in plasma and subcutaneous adipose tissue

Marker	PD group	PD-EX group
Plasma CRP, nmol/l		
Baseline	11 (6, 27)	13 (6, 23)
Change 0–12 weeks	−2 (−9, 1)	−3 (−8, 0)*
*IL6* in s.c. adipose tissue, relative expression of mRNA		
Baseline	0.81 (0.42, 1.16)	0.79 (0.61, 1.17)
Change 0–12 weeks	0.15 (−0.45, 0.62)	−0.14 (−0.42, 0.01)
*TNFα* in s.c. adipose tissue, relative expression of mRNA		
Baseline	2.19 (1.79, 3.03)	2.45 (1.89, 3.81)
Change 0–12 weeks	−0.06 (−0.53, 0.35)	−0.70 (−1.02, 0.32)

Data are reported as median (interquartile range)

*p < 0.05 for the within-group change over time from baseline to 12 weeks

## Discussion

This intervention with a Paleolithic diet in overweight individuals with type 2 diabetes showed decreased ectopic lipid accumulation in liver and soleus muscle, as well as improved peripheral insulin sensitivity. On a group level, the addition of combined resistance and aerobic exercise training to the diet intervention reduced the effect on muscle and liver fat content. This was due to the considerable heterogeneity in response to exercise. Decreased liver fat during the intervention was strongly associated with reduction in plasma fetuin-A levels in both intervention groups. This was linked to an improvement in adipose tissue insulin sensitivity.

Current guidelines for non-alcoholic fatty liver disease (NAFLD) recommend lifestyle interventions involving diet and exercise to decrease liver fat [33]. However, earlier studies suggest a complex relationship between liver fat and the effect of lifestyle interventions. Two short-term studies showed that weight reduction by a low-carbohydrate diet effectively reduced liver fat [4, 5]. We found a reduction in liver fat after a Paleolithic diet with a moderately reduced carbohydrate content. We anticipated that our combined intervention in individuals with type 2 diabetes would show an additional fat-decreasing effect on the liver, as a recent meta-analysis concluded that exercise reduces hepatic fat content [34]. Unexpectedly, three individuals in the PD-EX group showed a clear increase in liver fat. After exclusion of these three individuals, we found that liver fat decreased significantly in both study groups, with no difference between groups, while all other comparisons were unaltered. There may be several explanations for the unexpected response regarding liver fat in some individuals, including a prolonged exercise-induced increase in liver fat in some individuals or increased metabolic flexibility. The three individuals whose liver fat increased during 12 weeks of exercise thus showed decreased or unchanged inflammation (plasma CRP levels), triacylglycerol levels and liver enzymes, indicating improved metabolic health in this subgroup. A third possibility is that the participants did not refrain from exercise for 48 h before the examination as they were supposed to, leading to a decrease in muscle fat and an increase in liver fat.

In healthy people with normal weight and in overweight individuals, an acute bout of aerobic exercise immediately increases liver fat [35, 36]. This exercise-induced increase in liver fat seems to be mainly due to the rise in plasma NEFA during and after exercise [35, 36]. All participants in our study were told to refrain from exercise for 48 h prior to liver fat examination. A possible explanation for the conundrum could be that an exercise-induced increase in liver fat may last longer than 2 days in some individuals.

Lipolysis and inflammation are closely linked [37]. During our intervention, decreasing *TNFα* expression in adipose tissue was associated with decreasing plasma NEFA levels. Moreover, we found an association between liver fat and suppressibility of NEFA production, highlighting the importance of plasma NEFA concentration for hepatic lipid content. In NAFLD, most triacylglycerols in the fatty liver originate from plasma NEFA, and most plasma NEFA originate from adipose tissue [38]. Plasma NEFA uptake in liver cells and esterification into hepatic triacylglycerols are insulin-independent, depending only on the plasma NEFA concentration [39]. Notably, the percentage of plasma NEFA taken up by the liver remains constant both during and after exercise [40].

Plasma NEFA concentrations are also important for IMCL content, and we found that fasting plasma NEFA levels were closely associated with IMCL content of the tibialis anterior muscle. In healthy individuals, IMCL is used as an energy substrate during exercise and is replenished during recovery. Endurance athletes who performed 3 h of cycling exercise had a 20% decrease in IMCL content in the legs and a simultaneous 38% increase in IMCL content in the non-exercising arms [22]. Importantly, obesity and type 2 diabetes are associated with a lack of this dynamic response to exercise, which may be related to continuously increased plasma NEFA levels [23]. However, if adipose tissue lipolysis is reduced with a nicotinic acid analogue, plasma NEFA levels are reduced and the decrease in IMCL content during one bout of exercise is more pronounced [41].

Another contributing factor to the heterogeneous response in the combined diet and exercise intervention is the intake of carbohydrates. Suppression of adipose tissue lipolysis through insulin administration or carbohydrate ingestion leads to a reduction in plasma NEFA levels. Indeed, glucose ingestion during exercise causes plasma NEFA levels to decrease below fasting levels during and after exercise [36, 42]. Accordingly, glucose supplementation during and after cycling prevents an increase in liver fat during the recovery phase [36]. Furthermore, studies with isoenergetic diets show a decrease in liver fat only with high-carbohydrate diets, not with high-fat diets [6, 7]. Although energy intake was ad libitum in our study, participants reported decreases in carbohydrate and total energy intake. More detailed studies of macronutrient intake in relation to exercise are therefore of interest regarding the effects on hepatic lipid content.

Fetuin-A is secreted mainly from liver and adipose tissue and is elevated in type 2 diabetes and NAFLD [17, 43, 44]. Circulating fetuin-A levels have been associated with severity of liver steatosis, independently of insulin resistance, and with non-alcoholic steatohepatitis [45, 46]. Our results showed a strong association between fetuin-A levels and changes in liver fat content. Changes in circulating fetuin-A levels were also associated with the ability to suppress NEFA production. Since fetuin-A can be secreted by both hepatocytes and adipocytes, it remains unclear whether fetuin-A secreted by the liver influences adipose tissue or the other way round.

The PD group increased insulin clearance, which might have been due to the improvement in liver fat content. Hepatocytes thus show impaired insulin clearance when loaded with triacylglycerols in vitro [47], and liver fat is inversely related to insulin clearance in vivo [48].

Improvement of hepatic insulin sensitivity was less pronounced in our intervention: only if normalised by insulin, the PD group increased hepatic insulin sensitivity. This may relate to the relatively well-preserved hepatic insulin sensitivity in our study cohort. In most studies, diet-induced weight loss in people with type 2 diabetes causes an increase in hepatic insulin sensitivity, but some authors report it unchanged [49, 50].

A limitation of our study is that the insulin dose might have been too high to detect changes in hepatic insulin sensitivity. Moreover, target plasma glucose during the euglycaemic clamp was 8 mmol/l, which may not represent euglycaemia, especially after the intervention when fasting glucose was normalised. A lower insulin dose and a lower glucose target during the clamp studies might have detected more subtle changes in hepatic insulin sensitivity. Indeed, we have previously demonstrated an improvement in HOMA-IR after 5 weeks and 6 months following a Paleolithic diet in healthy overweight participants [8, 9].

Another limitation is that we had to exclude soleus muscle measurements from three participants because we could not separate the intramyocellular and extramyocellular lipid signals. This is a known technical difficulty related to the fact that soleus muscle fibres are not aligned in parallel to the main magnetic field. This shortage of data limits our ability to draw conclusions regarding the effects of the intervention on different skeletal muscle types. Finally, analyses of gene variants that may influence liver fat accumulation, e.g. *PNPLA3*, are of interest in future intervention studies.

In conclusion, our results indicate that an exercise intervention is associated with a heterogeneous response in liver fat content in obese individuals with type 2 diabetes, despite improved metabolic health. Further studies are needed to understand how exercise changes liver fat and hepatic insulin sensitivity in relation to energy balance and macronutrient intake among individuals with obesity and type 2 diabetes.

## Electronic supplementary material

ESM (PDF 158 kb)

## Data Availability

Data are available on request from the authors.

## Abbreviations

ALT: Alanine aminotransferase
AST: Aspartate aminotransferase
CRP: C-reactive protein
EGP: Endogenous glucose production
FFM: Fat-free mass
IMCL: Intramyocellular lipid
NAFLD: Non-alcoholic fatty liver disease
PD group: Paleolithic diet group
PD-EX group: Paleolithic diet and exercise group
