# Supplementary material for: A heterogeneous response of liver and skeletal muscle fat to the combination of a Paleolithic diet and exercise in obese individuals with type 2 diabetes: a randomised controlled trial
Source: Diabetologia. 2018 Apr 26;61(7):1548–59. doi: 10.1007/s00125-018-4618-y (PMC6445456; doi:10.1007/s00125-018-4618-y)
Supplement: Supplementary file 1 — (PDF 158 kb) [file 125_2018_4618_MOESM1_ESM.pdf]

ESM Methods

Liver fat, and fat in the tibialis anterior/soleus muscle Participants were instructed to avoid physical exercise for 48 h prior to examination. The proportions of fat in the liver, and soleus and tibialis anterior muscle were determined using proton magnetic resonance spectroscopy (^{1}H-MRS) with a Discovery MR750 3.0T GE scanner. Liver scans were performed using an 8-element cardiac coil, and calf muscle scans were performed using a quadrature knee coil. The liver spectroscopic volume was placed in the right lobe of the liver. For liver measurements, respiratory-triggered magnetic resonance spectra were acquired with the point-resolved spectroscopy sequence, using a repetition time (TR) of two respiratory intervals (at least 4500 ms), and an echo time (TE) of 28 ms. For the calf muscles (soleus and tibialis anterior), spectra were acquired with a 5000-ms TR and a 28-ms TE. For each spectrum, 64 signal averages were acquired from a volume element of $20 \times 20 \times 20$ mm^3. A total of 1024 data points were acquired over a 2000-Hz spectral width. In each case, two spectra were acquired: one with and one without pre-saturation of the water resonance. All MR spectra were processed and analyzed using the software package LCModel version 6.2-1P by S. W. Provencher. Lipid content was calculated as the sum of the lipid methylene resonances at 1.3 ppm and 1.6 ppm. The LCModel concentration table of lipid spectra reports liver fat concentrations as resonance areas divided by the unsuppressed water resonance area; thus, the lipid/water ratios were converted to percent lipid content using the following formula: lipid content (%) = 100 * lipid / (lipid + water) [1]. IMCL is measured as mM of CH_2 groups and normalised by creatine concentration of the muscle.

Gene expression After an overnight fast a needle biopsy of superficial subcutaneous adipose tissue was taken under local anesthesia from the lower abdomen. Adipose tissue was immediately frozen in liquid nitrogen and stored at −80 °C. Total RNA was extracted from subcutaneous adipose tissue biopsies using RNeasy® Lipid Tissue Mini kit (Qiagen, GmbH, Hilden, Germany) and RNA was reversed transcribed using TaqMan® reverse transcription reagents (Applied Biosystems, Foster City, CA, USA) as previously described [2] Relative quantification real-time PCR was performed on ABI Prism® 7000 Sequence Detection System (Applied Biosystems, Foster City, CA, USA) using Universal PCR Master Mix 2X (Roche Molecular Systems) and TaqMan gene expression assays (Applied Biosystems) for *TNFα* (Hs00174128_m1), *IL-6* (Hs00985639_m1) and *LRP10* (Hs00204094_m1). Reference genes was evaluated by comparing *PPIA* (Hs999999904_m1) and *LRP1019* on the study cohort using the NormFinder algorithm and *LRP10* appeared to be the most suitable gene and accordingly expression levels of the target genes were normalized to *LRP10*.

ESM Table 1 Results of the intervention without the three outliers of the Paleolithic diet + Exercise group that increase liver fat

	Paleolithic diet (n=13)	Paleolithic diet + Exercise group (n=10, without outliers)	Outlier 1	Outlier 2	Outlier 3
Liver fat, %					
Baseline	20 (14 – 28)	13 (6 − 21)	11	11	26
Change 0−12 weeks	−13 (−17, −9)**	−7 (−12, −3)*	10	6	7
Soleus muscle IMCL					
Baseline	19 (13 – 24)	13 (8 − 32)	31	26	33
Change 0−12 weeks	−9 (−12, −3)**	−2 (−3, 2)††	−7	−7	−20
Age, years	60 (54 – 64)	61 (58 − 67)	61	66	58
Diabetes duration, years	3 (2 – 6)	3 (1 − 7)	1	8	8
VO_2max, L/min					
Baseline	2.2 (2.0 – 2.6)	2.2 (1.7 – 3.0)	1.9	1.6	2.8
Change 0−12 weeks	−0.1 (−0.1, −0.3)*	0.1 (0.0, 0.4)*† †	0.2	0.2	0
Body weight, kg					
Baseline	90.0 (83.3 – 103.2)	97 (79 − 105)	84	84	126
Change 0−12 weeks	−7.1 (−9.8, −5.6)***	−7 (−9, −6)**	−5	−8	−14
BMI, kg/m^2					
Baseline	31.4 (29.4 − 33.7)	31.4 (28.6 – 32.9)	37.2	29.1	38.5
Change 0−12 weeks	−2.4 (−3.1, −1.8)***	−2.3 (−3.0, −2.0)**	−2.2	−2.8	−4.2
Fat mass, kg					
Baseline	34.4 (30.1, 37.9)	32.6 (28.9, 36.3)	35.8	37.3	42.7
Change 0−12 weeks	−5.7 (−8.2, −4.0)***	−6.0 (−8.6, −5.2)**	−3.8	−6.9	−10.1
HbA1c, mmol/mol					
Baseline	55 (48 − 58)	55 (49 – 58)	52	57	71
Change 0−12 weeks	−11 (−15, −5)**	−9 (−16, −5)*	−11	−19	−32
HbA1c, %					
Baseline	7.2 (6.5 − 7.5)	7.2 (6.6 – 7.5)	6.9	7.4	8.6
Change 0−12 weeks	−1.0 (−1.4, −0.5)**	−0.9 (−1.5, −0.5)*	−1.0	−1.8	−2.9
Fasting P-glucose, mmol/l					
Baseline	8.0 (6.9 – 8.5)	8.5 (7.5 – 10.1)	8.2	12.3	10.4
Change 0−12 weeks	−0.9 (−1.8, −0.1)*	−1.2 (−2.3, −0.8)*	−2.0	−4.4	−4.0

P-triacylglycerols, mmol/l					
Baseline	2.4 (1.4 – 3.1)	1.7 (1.1 − 2.2)	1.0	0.9	3.6
Change 0−12 weeks	−0.6 (−1.8, −0.2)*	−0.5 (−0.9, −0.2)**	0.0	−0.2	−2.5
P-NEFA, mmol/l					
Baseline	0.60 (0.53 – 0.78)	0.85 (0.65 − 0.93)	0.68	0.81	0.64
Change 0−12 weeks	0.03 (−0.02, 0.20)	−0.02 (−0.10, 0.15)	−0.09	0.32	−0.14
P-AST, μkat/l					
Baseline	0.59 (0.53 – 0.71)	0.53 (0.50 − 0.65)	0.95	0.51	0.63
Change 0−12 weeks	−0.05 (−0.14, 0.14)	0.06 (−0.11, 0.27)	−0.28	0.00	−0.25
P-ALT, μkat/l					
Baseline	0.67 (0.53 – 0.91)	0.51 (0.46 − 0.69)	1.63	0.49	0.67
Change 0−12 weeks	−0.12 (−0.34, −0.08)**	−0.01 (−0.13, 0.11) †	−0.67	−0.06	−0.23
Plasma fetuin-A, μg/ml					
Baseline	455 (414 – 558)	423 (370 – 560)	433	128	416
Change 0−12 weeks	−44 (−87, −3)*	−5 (−80, 7)	57	264	73
Energy intake, kJ/day					
Baseline	8330 (6204 – 10778)	6517 (5477 – 8037)		9615	11389
Change 0−12 weeks	–1377 (–3284, –1025)**	–2155 (–2766, –531)**		–3644	–657
Total energy expenditure, kJ/day					
Baseline	12619 (11129 – 13933)	12519 (11355 – 16539)	9627	8761	17364
Change 0−12 weeks	–954 (–1485, –285)*	–1305 (–2360, 611)		1201	–5259
Resting energy expenditure, kJ/day					
Baseline	6791 (6184 – 7243)	7272 (5653 – 7904)	5561	5565	10109
Change 0−12 weeks	–510 (–774, –188)**	–364 (–699, –38)*		–540	–2192
Physical activity energy expenditure, kJ/day					
Baseline	4276 (3615 – 5519)	4874 (3728 – 7544)	3192	2402	5678
Change 0−12 weeks	–117 (–870, 372)	–644 (–1623, 1678)	791	1632	–2586
Protein, g/day					
Baseline	80 (69 – 95)	73 (58 – 92)		108	136
Change 0−12 weeks	5 (−17, 23)	1 (−14, 12)		−29	15
Protein, g kg^{-1} day^{-1}					
Baseline	0.85 (0.66 – 1.14)	0.75 (0.73 – 0.84)		1.29	1.08

Change 0−12 weeks	0.10 (−0.09, 0.35)	0.06 (−0.10, 0.23)	−0.25	0.26
Carbohydrate, g/day				
Baseline	204 (148 – 280)	161 (148 – 186)	186	303
Change 0−12 weeks	−89 (−122, −49)**	−84 (−117, −62)**	−115	−117
Total fat, g/day				
Baseline	84 (58 – 115)	58 (44 – 78)	118	98
Change 0−12 weeks	−12 (−38, 8)	−10 (−30, 20)	−35	31
Saturated fatty acids, g/day				
Baseline	31 (21 – 48)	23 (19 – 29)	48	36
Change 0−12 weeks	−14 (−33, −5)**	−11 (−19, −5)**	−25	−11
Monounsaturated fatty acids, g/day				
Baseline	32 (25 – 41)	21 (15 – 31) †	44	37
Change 0−12 weeks	4 (−16, 14)	5 (−7, 17)	−4	33
Polyunsaturated fatty acids, g/day				
Baseline	11 (9 – 14)	8 (6 – 12)	18	16
Change 0−12 weeks	1 (−5, 5)	1 (−4, 7)	−3	8
Protein, E%				
Baseline	17 (14 – 19)	18 (16 – 21)	19	20
Change 0−12 weeks	7 (4, 11)**	7 (4, 14)**	3	4
Carbohydrate, E%				
Baseline	41 (38 – 46)	47 (31 – 49)	32	45
Change 0−12 weeks	−10 (−18, −3)*	−13 (−22, −6)**	−13	−16
Total fat, E%				
Baseline	40 (36 – 41)	32 (30 – 40)	46	33
Change 0−12 weeks	6 (−6, 11)	8 (0, 15)	6	13
Saturated fatty acids, E%				
Baseline	15 (13 – 18)	13 (11 – 16)	19	12
Change 0−12 weeks	−5 (−8, −3)**	−4 (−9, −1)**	−4	−3
Monounsaturated fatty acids, E%				
Baseline	16 (14 – 17)	12 (10 −16)	17	12
Change 0−12 weeks	5 (−3, 11)	10 (3, 12)*	8	12
Polyunsaturated fatty acids, E%				

Baseline	5.0 (4.7 – 6.4)	5.1 (3.7 – 6.4)		7	5
Change 0−12 weeks	2.1 (−0.5, 3.6)*	3.3 (0.1, 3.9)*		3	3
Rate of disappearance, mg kg^{-1} min^{-1}					
Baseline	3.79 (2.95 – 4.23)	3.9 (2.9 − 5.5)	4.1	3.9	3.2
Change 0−12 weeks	2.05 (0.32, 3.59)*	1.0 (0.5, 3.1)*	0.8	2.8	2.2
Rate of disappearance/insulin, μg kg^{-1} min^{-1} per mIU/l					
Baseline	34.2 (28.6 − 49.2)	51.7 (33.1 – 75.8)	35.1	61.4	46.2
Change 0−12 weeks	28.9 (11.8, 61.5)**	12.2 (0.4, 25.2)*	14.6	27.1	33.5
Endogenous glucose production, mg kg^{-1} min^{-1}					
Baseline	1.81 (1.56 − 1.99)	1.78 (1.50 – 2.69)	1.48	1.92	1.91
Change 0−12 weeks	0.04 (−0.06, 0.55)	0.19 (−0.33, 0.70)	0.11	−0.24	−0.06
Suppression of endogenous glucose production, %					
Baseline	96 (83 – 128)	100 (85 − 131)	36	114	116
Change 0−12 weeks	13 (−10, 44)	14 (−43, 34)	0	−25	96
Suppression of endogenous glucose production/insulin, % per mIU/l					
Baseline	0.98 (0.77 − 1.36)	1.39 (1.04 – 1.72)	0.31	1.81	1.67
Change 0−12 weeks	0.22 (−0.03, 0.91)*	−0.02 (−0.44, 0.44)	0.06	−0.63	1.43
Suppression of NEFA, %					
Baseline	88 (80 – 93)	89 (85 − 93)	92	93	69
Change 0−12 weeks	3.4 (1.3, 5.7)*	3.5 (0.2, 6.4)*	0	2	21
Suppression of NEFA/insulin, % per mIU/l					
Baseline	0.83 (0.74 − 1.08)	1.13 (1.01 −1.26) †	0.78	1.47	0.99
Change 0−12 weeks	0.14 (0.03, 0.31)**	0.09 (−0.03, 0.19)	0.14	−0.22	0.33
P-CRP, nmol/l					
Baseline	11 (6 – 27)	13 (6 – 24)	15	15	6
Change 0−12 weeks	−2 (−9, 1)	−3 (−10, 0)*	−5	−7	0
IL6 in s.c. adipose tissue, relative expression of mRNA					
Baseline	0.81 (0.42 – 1.16)	0.78 (0.52 – 1.08)	0.82	3.93	0.82
Change 0−12 weeks	0.15 (−0.45, 0.62)	−0.13 (−0.30, −0.01)	0.09	−3.42	−0.46
TNFα in s.c. adipose tissue, relative expression of mRNA					
Baseline	2.19 (1.79 – 3.03)	2.26 (1.75 – 3.34)	3.75	3.88	2.45
Change 0−12 weeks	−0.06 (−0.53, ,0.35)	−0.61 (−0.92, 1.04)	−2.05	0.18	−1.12

Data are reported as median (interquartile range) and individuals results for three subjects. *$p < 0.05$, **$p < 0.01$, ***$p < 0.001$ for the within-group change over time from baseline to 12 weeks. †$p < 0.05$, ††$p < 0.01$ between the Paleolithic diet group and the Paleolithic diet + Exercise group minus 3 outliers. Abbreviations: P-ALT, Plasma-Alanine aminotransferase. P-AST, Plasma-Aspartate aminotransferase. P-CRP, Plasma-C-reactive protein. E%, energy percent. IMCL, intramyocellular lipids. P-NEFA, Plasma-non-esterified fatty acids.

References

1. Ryysy L, Hakkinen AM, Goto T, et al. (2000) Hepatic fat content and insulin action on free fatty acids and glucose metabolism rather than insulin absorption are associated with insulin requirements during insulin therapy in type 2 diabetic patients. Diabetes 49: 749-758
2. Alvehus M, Simonyte K, Andersson T, et al. (2012) Adipose tissue IL-8 is increased in normal weight women after menopause and reduced after gastric bypass surgery in obese women. Clin Endocrinol (Oxf) 77: 684-690
